# Serum Neuron-Specific Enolase as a Biomarker of Neonatal Brain Injury—New Perspectives for the Identification of Preterm Neonates at High Risk for Severe Intraventricular Hemorrhage

**DOI:** 10.3390/biom14040434

**Published:** 2024-04-03

**Authors:** Dimitra Metallinou, Grigorios Karampas, Maria-Loukia Pavlou, Maria-Ioanna Louma, Aimilia Mantzou, Antigoni Sarantaki, Christina Nanou, Kleanthi Gourounti, Maria Tzeli, Nikoletta Pantelaki, Evangelos Tzamakos, Theodora Boutsikou, Aikaterini Lykeridou, Nicoletta Iacovidou

**Affiliations:** 1 Department of Midwifery, University of West Attica, 12243 Athens, Greece; mariloupav@gmail.com (M.-L.P.); esarantaki@uniwa.gr (A.S.); nanouxv@uniwa.gr (C.N.); kgourounti@uniwa.gr (K.G.); mtzeli@uniwa.gr (M.T.); npantelaki@uniwa.gr (N.P.); tzamvagpal@uniwa.gr (E.T.); klykeridou@yahoo.gr (A.L.); 2 School of Medicine, National and Kapodistrian University of Athens, 11527 Athens, Greece; 3 Second Department of Obstetrics and Gynaecology, Aretaieio Hospital, National and Kapodistrian University of Athens, 11528 Athens, Greece; karampasgr@yahoo.gr; 4 Department of Biochemistry & Biotechnology, University of Thessaly, 41500 Larissa, Greece; mariannalouma@yahoo.gr; 5 Division of Endocrinology, Diabetes and Metabolism, First Department of Pediatrics, Medical School, Aghia Sophia Children’s Hospital, National and Kapodistrian University of Athens, 11528 Athens, Greece; amantzou@med.uoa.gr; 6 Department of Neonatology, School of Medicine, Aretaieio Hospital, National and Kapodistrian University of Athens, 15772 Athens, Greece; theobtsk@gmail.com (T.B.); niakobid@med.uoa.gr (N.I.)

**Keywords:** neuron-specific enolase, serum, brain injury, preterm neonate, biomarkers, intraventricular hemorrhage, periventricular leukomalacia, neonatal intensive care unit

## Abstract

Neonatal brain injury (NBI) is a critical condition for preterm neonates with potential long-term adverse neurodevelopmental outcomes. This prospective longitudinal case–control study aimed at investigating the levels and prognostic value of serum neuron-specific enolase (NSE) during the first 3 days of life in preterm neonates (<34 weeks) that later developed brain injury in the form of either periventricular leukomalacia (PVL) or intraventricular hemorrhage (IVH) during their hospitalization. Participants were recruited from one neonatal intensive care unit, and on the basis of birth weight and gestational age, we matched each case (*n* = 29) with a neonate who had a normal head ultrasound scan (*n* = 29). We report that serum NSE levels during the first three days of life do not differ significantly between control and preterm neonates with NBI. Nevertheless, subgroup analysis revealed that neonates with IVH had significantly higher concentrations of serum NSE in comparison to controls and neonates with PVL on the third day of life (*p* = 0.014 and *p* = 0.033, respectively). The same pattern on the levels of NSE on the third day of life was also observed between (a) neonates with IVH and all other neonates (PVL and control; *p* = 0.003), (b) neonates with II–IV degree IVH and all other neonates (*p* = 0.003), and (c) between control and the five (*n* = 5) neonates that died from the case group (*p* = 0.023). We conclude that NSE could be an effective and useful biomarker on the third day of life for the identification of preterm neonates at high risk of developing severe forms of IVH.

## 1. Introduction

Neonatal brain injury (NBI) refers to damage or injury to the neonatal brain, typically occurring within the first 28 days of life. This can result from a variety of causes and have long-term consequences for the child’s development. Neonatal brain injury is a complex and multifactorial condition, and it can manifest in different ways, depending on the underlying cause and the extent of the damage. Some common causes are perinatal asphyxia, metabolic disorders, and traumatic injuries during assisted vaginal delivery [1,2], while relevant risk factors include, indicatively, extreme prematurity, absent antenatal steroid and magnesium sulfate (MgSO_4_) treatment, pneumothorax, and inherited thrombophilia [3,4,5]. Specifically for preterm neonates, it is estimated that periventricular leukomalacia (PVL) and intraventricular hemorrhage (IVH) are prevalent despite the advancements in perinatal medicine and clinical management [6,7]. The neonatal outcome varies depending on the severity and location of the brain injury, as well as the promptness and effectiveness of medical intervention [8]. Advances in biomarkers for NBI have the potential to revolutionize the way healthcare professionals diagnose, monitor, and manage ΝΒΙ.

A growing body of literature has investigated biomarkers in NBI. Nevertheless, it remains a challenge to diagnose brain damage in neonates in the early neonatal period. Considering the complexity of NBI, it seems challenging that a single biomarker can represent the entire picture of an injured brain. Therefore, experts are focusing on multimodal biomarker panels and their interpretation using artificial intelligence, which may provide a more comprehensive picture of NBI, improving diagnostic accuracy and predictive power [9]. In recent years, novel biomarkers [10,11] have been significantly correlated with the prediction of NBI risk and the rapid assessment in various biological fluids [5,12,13]. Such biomarkers may also lead to early identification and diagnosis of NBI at its earliest stages, profiling, and personalized treatment strategies. Individualized care, along with brain-focused clinical practices for preterm neonates, should form the primary goals for healthcare professionals in neonatal intensive care units (NICUs) in order to reduce the severity of the injury and improve the neurodevelopmental outcomes of the affected neonates [11,14].

Neuron-specific enolase (NSE), a promising biomarker for NBI [15,16], is an enzyme that is primarily found in neurons and peripheral neuroendocrine cells [17]. It is also known as gamma-enolase and enolase 2 (ENO2). NSE is involved in glycolysis, a metabolic pathway that generates energy in the form of adenosine triphosphate (ATP) within cells. In neurons, NSE plays a crucial role in energy production, and it is often used as a marker to identify and measure neuronal or neuroendocrine cell damage or injury [18,19]. Therefore, measuring the levels of NSE in the blood can be a valuable tool in clinical settings. It is important to note, though, that NSE is not specific to any particular neurological disorder and must be interpreted in the context of other clinical information and diagnostic tests to provide a comprehensive understanding of the patient’s condition [19].

NSE has already been explored in various biological fluids since the 1980s. In their cutting-edge paper of 1986, Gotoh et al. [20] reported that NSE in urine may be a valuable marker for monitoring the effectiveness of therapy in patients with neuroblastoma, while later in the early 2000s, Wijnberger et al. [21] investigated the potential association of NSE in cord blood, amniotic fluid, and placental tissue with perinatal damage. A recent review of the literature [22] revealed that an increasing number of studies have found that NSE concentrations are significantly increased in hypoxic ischemic encephalopathy (HIE) and neonates with perinatal asphyxia in comparison to healthy controls. HIE and long-term neurodevelopmental outcomes have been the main focus of previous studies [22,23,24], so few researchers have investigated NSE levels in preterm neonates [25,26,27]. Due to the great heterogeneity in the studied population among these studies, the role of NSE in the field of NBI remains unclear. Thus, in the present study, we aimed to investigate whether serum NSE levels during the first 3 days of life in preterm neonates (<34 weeks) (a) differ significantly between controls and neonates with NBI (case group) and (b) have a predictive value regarding the early identification of high-risk preterm neonates to develop brain injury, resulting in severe adverse neonatal outcomes such as death, seizures, and/or hypertonia, with the prospect of providing further evidence into the growing body of scientific literature.

## 2. Materials and Methods

This prospective longitudinal case–control study is part of a larger research protocol that investigates NBI biomarkers in preterm neonates [5,19,28]. This study is based on methods previously published and is outlined below:

The study recruited preterm (<34 weeks) neonates from a Neonatal Intensive Care Unit (NICU) located in a single tertiary hospital. Exclusion criteria have been previously reported [5]. Umbilical catheters placed after birth or peripheral vessels were used to collect the serial blood samples during the first three days of life, one sample per day. On the first day of life, the blood sample was obtained when the neonate was admitted to the NICU immediately after birth. Blood specimens were collected in pediatric gel and clot activator tubes (microtainers of 400 microlitres) (BD, Franklin Lakes, NJ, USA). Samples were left to clot for two hours at room temperature and then centrifuged. Serum remaining after the clinical routine laboratory tests was used for the measurements of NSE and was stored in aliquots at −35 °C until analyzed. Hemolytic specimens were rejected.

Considering all the head ultrasound scans (HUS) being performed during hospitalization, NBI was classified at discharge when neonates were allocated to the case or control group. Cases were diagnosed with NBI in the form of either PVL or IVH, while neonates with hypoxic ischemic encephalopathy (HIE) were excluded from the study. HUS patterns followed the European Standards of Care for Newborn Health (ESCNH) [29], IVH was classified according to Papile et al. [30], and periventricular leukomalacia, according to de Vries et al. [31].

This study was conducted in accordance with the ethical principles stated in the Helsinki Declaration. Written informed consent was obtained from all neonates’ parents included in the study. Ethics approval was granted by the Committee of “Aretaieio” University Hospital (IRB R.No.: B-216/13-10-2016/APPROVAL NUMBER-ID: KM140657).

The research team reviewed obstetric and neonatal records to explore if NBI was associated with certain perinatal factors and outcomes. By using an automatic coding system, the database ensured anonymization/deidentification for all participants (mothers and neonates). The NSE concentrations were determined using a kit available from a commercial source (SEA537Hu ELISA kit from Cloud-Clone Corp., Wuhan, China). In accordance with the manufacturer’s data, the lowest detection limit was 0.065 ng/mL, and the precision, as estimated by the total CV (%), was <5.7%. Values < 0.065 ng/mL were reported as zero.

The data analysis was conducted using IBM SPSS statistics version 23 (IBM Corporation, Somers, NY, USA). Statistical analysis and cross-checking were performed by study personnel. This wider research protocol bases sample size calculations on S100B levels, which are considered the “gold standard” for NBI biomarkers. It was estimated that 5–24 neonates would offer sufficient statistical power. Several characteristics were compared between the mothers and neonates to ensure successful matching and identify differences between them. Pearson’s chi-square test (X2) was used to compare qualitative data between the two groups. The one-sample Kolmogorov–Smirnov test was used to test the normality of the concentration of NSE and the other quantitative parameters. The NSE concentration and other quantitative parameters were compared between groups using parametric Student’s *t*-tests, as the one-sample Kolmogorov–Smirnov test revealed a normal distribution of the parameters included in the statistical analysis. A comparison of NSE levels within the two groups during the first three days of life was performed by a one-way analysis of variance (ANOVA) test for normally distributed parameters. Furthermore, subgroup analyses were conducted among control neonates and neonates with either PVL or IVH in order to determine if NSE levels varied in different forms of NBI. The five neonates that died from the case group were also compared to controls and the remainder of the cases in order to determine whether NSE is altered by such a severe adverse neonatal outcome during the first three days of life. As a final step, a multivariate logistic regression model was used to examine the predictive value of serum NSE regarding NBI. The presence of II–IV degree IVH at discharge from the NICU was set as an outcome, using as predictive variables serum NSE levels during the first three days of life. A probability level of less than or equal to 0.05 was considered significant.

## 3. Results

**Figure 1:**
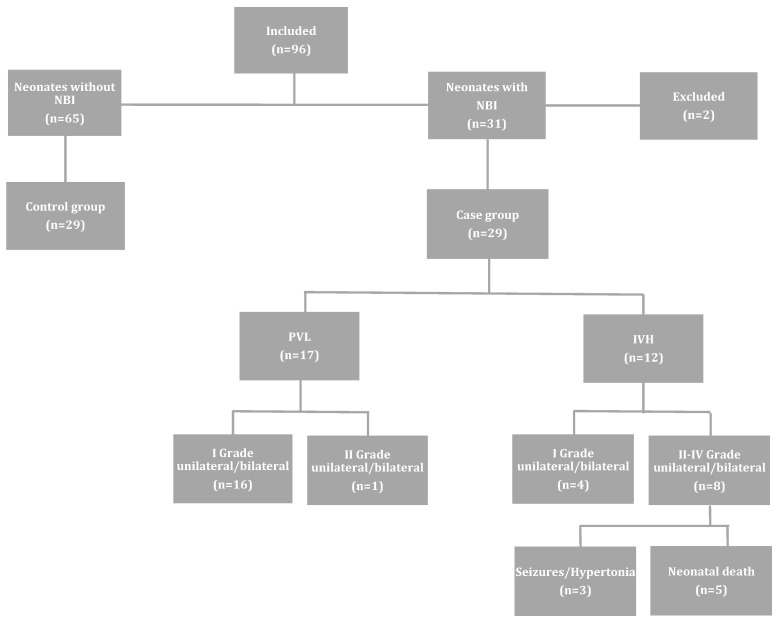
Flowchart of the studied population and perinatal outcome. NBI: neonatal brain injury; IVH: intraventricular hemorrhage; PVL: periventricular leukomalacia. Reprinted from [5], with permission from Elsevier.

Ninety-six (*n* = 96) neonates met the inclusion criteria and were eventually enrolled in the study. Sixty-five (*n* = 65) of these neonates did not develop NBI, while the remaining thirty-one (*n* = 31) were complicated by PVL (*n* = 17), IVH (*n* = 12), and HIE (*n* = 2). The latter two were excluded from the study. Therefore, the case group was comprised of twenty-nine (*n* = 29) neonates, and subsequently, twenty-nine (*n* = 29) neonates with normal HUS were matched one by one to the cases, taking into account the same gestational age (within one week) and similar birth weight (Figure 1). Of the 12 neonates that developed IVH, 8 were diagnosed with II–IV degree brain injury, from which 5 died. These neonatal deaths occurred more specifically on day 2 (*n* = 1), day 3 (*n* = 1), day 4 (*n* = 1), day 9 (*n* = 1) and day 12 (*n* = 1).

Maternal and neonatal characteristics and laboratory findings of the control and the case group have been extensively described previously [5]. In short, maternal demographic and clinical characteristics did not differ between the case and control groups. As for neonatal characteristics and laboratory findings, when compared to control neonates, neonates in the case group had significantly lower admission pH and white blood cell counts. The incidence of necrotizing enterocolitis was higher in control neonates. The incidence of seizures and death, as well as the admission base deficit and concentration of lactate acid, were higher in the case group. Finally, no difference was observed either in the length of stay in the NICU or therapeutic interventions during hospitalization between the two groups, including the use of surfactants, inotropes, caffeine, and the need for cardiopulmonary resuscitation.

The serum levels of NSE in the case and control group were comparable at 84/87 (96.5%) of the time points desired. The missing data were due to inadequate serum remaining after the standard routine exams were completed and due to neonatal death in the case group. The mean ± standard deviation (SD) of NSE levels in the case and control groups is presented in Table 1. A significant difference was observed between admission and the third day of life in the control group, with neonates presenting higher levels of NSE on the first day and showing a downward trend the following days (Table 1). On the contrary, no difference was observed in the case group during the first three days of life, with serum NSE showing relatively stable levels. Analysis between the two groups revealed that serum NSE did not differ significantly during the first three days of life (Table 1). Moreover, Pearson’s rank correlation coefficient (r) revealed that NSE levels did not show any significant correlation with gestational age in control neonates on the first day (r = −0.026, *p* = 0.899), second day (r = 0.081, *p* = 0.693), or third day (r = 0.307, *p* = 0.128) of life.

Further subgroup analysis included a comparison (a) among control neonates and neonates with either PVL or IVH (Table 2), (b) between neonates with IVH (*n* = 12) and all other neonates (*n* = 46) (Table 3), (c) between the eight (*n* = 8) neonates that developed II–IV degree of IVH and all other (*n* = 50) neonates (Table 4), and (d) between control and the five (*n* = 5) neonates that died from the case group (Table 5). That analysis revealed that neonates with IVH had significantly higher concentrations of serum NSE in comparison to controls and neonates with PVL on the third day of life (*p* = 0.014 and *p* = 0.033, respectively). The same pattern on the levels of NSE on the third day of life was also observed between (a) neonates with IVH and all other neonates (PVL and control; *p* = 0.003), (b) neonates with II–IV degree IVH and all other neonates (*p* = 0.003), and (c) control and the five neonates that died (*p* = 0.023).

**Figure 2:**
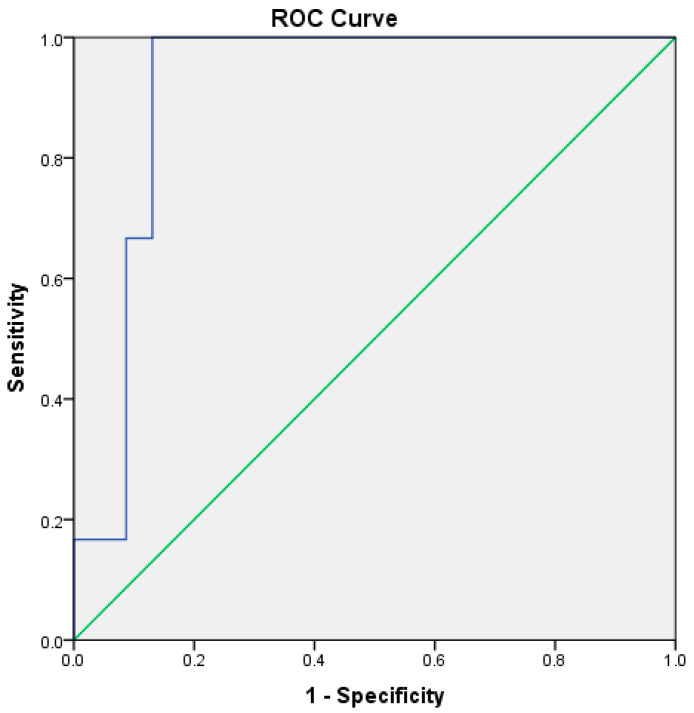
This ROC curve for serum neuron-specific enolase on the third day of life between neonates with II–IV degree intraventricular hemorrhage and all other neonates. Area under the curve: 91.3%, *p* = 0.001, 95% confidence interval: 83.4–99.2%.

A multivariate conditional logistic regression model was used to assess the predictive value of serum NSE during the first three days of life, with the presence or absence of II–IV-degree IVH at NICU discharge as the outcome and serum NSE levels during the first three days of life as the predictive variables. Using the forward stepwise conditional method, serum NSE on the first and third day of life were the best predictors for the adverse neonatal outcome (*p* = 0.002 for the model; *p* = 0.033 and *p* = 0.017 for serum NSE on the first and third day of life; *p* = 0.013 for the constant). More specifically, the receiver operating characteristic curve (ROC-curve) for serum NSE on the third day of life yielded a high predictive value, with the area under the curve at 91.3% (*p* = 0.001, 95%CI: 83.4–99.2%). According to the ROC-curve analysis for a cut-off value of 5.43 ng/mL, sensitivity was 100% and specificity was 87% (Figure 2).

## 4. Discussion

In the present study, we demonstrated that serum NSE levels during the first three days of life do not differ significantly between control and preterm neonates that will later develop NBI in the form of either PVL or IVH. This confirms previous findings in the literature, like the ones of Efstathiou et al. [27], who reported that blood NSE levels in preterm neonates less than 34 weeks of gestation, as our study population, in the first days of life (days 1, 3, and 9) showed no difference between the control and the case group. Nevertheless, a significant difference was observed on the 18th day of life, where NSE levels were higher in the brain injury group (PVL, IVH, and infarct) compared to controls. Probably, if we had extended our sample collection for more days, we might have found significant differences, so future similar studies should consider prolonging the longitudinal design. Moreover, this research group demonstrated that NSE levels in the control group significantly decreased from day 1 to 3, which is in complete agreement with the trend in our control group. It should be made clear, though, the differences between this study and ours. Firstly, the case group in Efstathiou’s study consisted of neonates who had undergone “severe perinatal stress with metabolic acidosis and developed CNS injury (IVH III or higher, PVL or infarct) [27,32], while the case group in our study was more representative of the general population of premature neonates as no neurologic morbidity was set as inclusion criteria. Secondly, Efstathiou measured the NSE in circulating progenitor cells (CPCs) and our team in serum, so there are significant differences in the context, implications, and technical approaches involved, which can affect the interpretation and application of the findings. More specifically, measuring biomarkers in CPCs can provide insights into regenerative processes, inflammation, and the body’s response to injury or disease at a cellular level. It is more about understanding cellular physiology, differentiation potential, and the body’s endogenous regenerative capabilities [33]. Biomarkers in serum generally reflect the overall health status, disease presence, or physiological changes at a systemic level. It is a more general measure, providing insights into the biochemical environment of the body, including the presence of diseases, infections, metabolic processes, and immune responses [34]. In addition, measuring biomarkers in CPCs often involves cell isolation from the blood, followed by cell culture or flow cytometry to identify specific cell populations and quantify biomarkers. This process can be technically challenging, time-consuming, and requires specific expertise to accurately identify and quantify biomarkers within these cells, which might not be readily available in all clinical settings. On the other hand, serum measurement is generally more straightforward and standardized, involving techniques such as enzyme-linked immunosorbent assay (ELISA), mass spectrometry, or other biochemical assays. These methods are widely used, easier to perform on a large scale, and typically require less specialized equipment and expertise compared to cell-based assays. Since serum is easier to obtain and analyze, this method is more commonly used in clinical practice. Lastly, the presence or level of certain biomarkers in CPCs is particularly relevant in research and clinical settings focused on regenerative medicine, whereas serum biomarkers are crucial for routine health screenings, disease diagnosis, monitoring of disease progression, and evaluating treatment efficacy. The choice between measuring a biomarker in CPCs or serum depends on the specific research or clinical question being addressed. Each approach has its own set of advantages, challenges, and applications, underscoring the importance of selecting the right method based on the specific context of the study or diagnostic need.

Furthermore, Attia et al. [22] investigated serum NSE levels in thirty-term neonates who developed symptoms and signs of HIE and concluded that NSE correlated significantly with grades II and III and neonates who suffered from neurological sequelae, a finding that lends support to our own results, although our studied population included neonates with different types of NBI. In addition, Paliwal et al. [35] explored serum NSE levels in late preterm and term neonates born with perinatal asphyxia and observed, firstly, a significant association between serum NSE levels and the severity of HIE and, secondly, a downward trend of NSE from day 1 to day 3 of life. Interestingly, similar variation over time is also confirmed by the present study, building a robust foundation of research findings.

To the best of our knowledge, no other studies, including any biological fluid except one [27], have studied NSE levels in preterm neonates in association with PVL and IVH. The present study, however, contributes new knowledge and value to the field as it applies to new contexts and conditions. Contrary to previous studies [25,26,27], our study recruited neonates with no neurologic morbidity at birth who developed later on NBI, and serum samples were collected at different time points (three samples sequentially on the first 3 days of life vs. one single sample or samples with intervals). We introduced new variables, including therapeutic interventions, numerous maternal/neonatal characteristics, and laboratory findings, minimizing by the matching procedure possible confounding factors. This may help to understand the complexity of the underlying pathophysiology more adequately and uncover aspects that were not addressed in previous works. Nevertheless, obtaining similar results with previous studies [27] adds robustness to existing evidence.

We feel strongly that early identification of preterm neonates at high risk of developing PVL or IVH is crucial for prompt and effective intervention, as these common lesions of the neonatal brain are still reported in high prevalence [6,7], despite the continuous progress in perinatal and neonatal care. The use of head ultrasound imaging is considered the gold standard for diagnosing but not predicting NBI, especially in premature neonates with PVL or IVH [36,37,38]. Also, MRI is capable of identifying mild-to-severe white matter injuries, which are linked to adverse neurodevelopmental outcomes, but its application in early life is limited [29,39]. Therefore, it is of great importance to have more blood-derived biomarkers that can contribute, in particular, to daily clinical practice, and based on the subgroup analysis presented above, NSE seems to be a useful biomarker on the third day of life for detecting neonates at high risk of developing severe IVH. However, we demonstrated that it is a biomarker with a late response in comparison to S100b [5], which was significantly elevated in the same studied population as early as the 1st day of life, especially on admission to the NICU. That limitation is crucial as the first days of hospitalization represent a rather critical period to implement pharmacological and non-pharmacological brain-focused clinical practices for neonates at high risk of neuronal injury [14]. Thus, the predictive value of serum NSE for brain injury in preterm neonates should be examined in combination with other specific biomarkers for brain injury, clinical findings, and routine laboratory tests long before the ultrasound findings are present, targeting early brain-saving interventions.

The present study integrates some important strengths, such as the prospective longitudinal methodology, the well-defined pathology in the case’s group, and the subgroup analysis based on the NBI subtype, which is reported for the first time in the literature. An additional strength of our study lies in the direct comparison of the levels and predictive value of NSE in preterm neonates at high risk of developing NBI to the levels and predictive value of S100B, as we have presented in our previous study [5]. As we have included in our analyses the same neonates in both the control and the case groups, our findings add substantially to our understanding of the NSE’s function and usefulness. Thus, we have demonstrated that S100B is an early biomarker for NBI in preterm neonates, already from the first day of life, while NSE seems to have a later response, presenting a predictive value from the third day of life. Nevertheless, it is plausible that a number of limitations might have influenced the results obtained. A major limitation is that both groups included a small number of neonates, and especially the case group had relatively few neonates who suffered severe outcomes, such as grade II–IV IVH or death. This could possibly explain why we failed to detect statistical significance between the two groups regarding their serum NSE levels during the first two days of life. However, in the present study, IVH cases had a higher grade of NBI in comparison to those with PVL, and this difference in severity of NBI was represented in the levels of NSE in the different sub-group analyses we performed, where neonates with IVH had a higher concentration of serum NSE compared to controls and neonates with PVL on the third day of life.

## 5. Conclusions

Neuron-specific enolase seems to represent a useful biomarker for the identification of preterm neonates at high risk of developing severe NBI and, more specifically, II–IV degree IVH long before the imaging findings are present. Nevertheless, due to its late response, it will have to be combined with other brain injury biomarkers with respect to providing an effective and applicable predictive model to everyday clinical practice. Thus, further larger prospective case–control studies are needed in order to precisely determine its predictive value and ideal cut-off levels in serum.

## Figures and Tables

**Table 1:** Concentrations of serum NSE (ng/mL) in the two studied groups during the first 3 days of life (mean ± standard deviation).

N	Control Group	Case Group	*p*-Value
	29	29	
Admission Mean (±SD)	5.91 (±3.68)	5.62 (±3.11)	0.904
2nd day Mean (±SD)	4.92 (±2.14)	5.12 (±2.8)	0.521
3rd day Mean (±SD)	3.86 (±1.68) ^1^	5.06 (±3.49)	0.124

^1^ *p* = 0.02, compared to admission in control neonates; SD: standard deviation.

**Table 2:** Concentrations of serum NSE (ng/mL) in control and neonates with either periventricular leukomalacia (PVL) or intraventricular hemorrhage (IVH) during the first 3 days of life (mean ± standard deviation).

N	Control Group	PVL	IVH	*p*-Value
	29	17	12	
Admission Mean (±SD)	5.91 (±3.68)	5.66 (±3.15)	5.57 (±3.21)	NS
2nd day Mean (±SD)	4.92 (±2.14)	4.19 (±2.29)	6.38 (±3.03)	NS
3rd day Mean (±SD)	3.86 (±1.68) ^1^	3.91 (±2.78)	6.62 (±3.87)	0.014 ^2^ 0.033 ^3^

SD: standard deviation; NS: not significant; ^1^ *p* = 0.02, compared to admission in control neonates; ^2^ between control and neonates with IVH; ^3^ between neonates with PVL and those with IVH.

**Table 3:** Concentrations of serum NSE (ng/mL) in neonates with intraventricular hemorrhage (IVH) and all other neonates during the first 3 days of life (mean ± standard deviation).

N	All Other (Controls and PVL)	IVH	*p*-Value
	46	12	
Admission Mean (±SD)	5.82 (±3.46)	5.57 (±3.21)	0.592
2nd day Mean (±SD)	4.65 (±2.21)	6.38 (±3.03)	0.083
3rd day Mean (±SD)	3.88 (±2.12)	6.62 (±3.87)	0.003

SD: standard deviation; PVL: periventricular leukomalacia.

**Table 4:** Concentrations of serum NSE (ng/mL) in neonates with II–IV degrees intraventricular hemorrhage (IVH) and all other neonates during the first 3 days of life (mean ± standard deviation).

N	All Other (Controls and PVL)	II–IV Degree IVH	*p*-Value
	50	8	
Admission Mean (±SD)	5.83 (±3.38)	5.34 (±3.65)	0.715
2nd day Mean (±SD)	4.81 (±2.19)	6.64 (±3.98)	0.146
3rd day Mean (±SD)	4.06 (±2.43)	7.52 (±3.57)	0.003

SD: standard deviation; PVL: periventricular leukomalacia.

**Table 5:** Concentrations of serum NSE (ng/mL) in control and neonates with intraventricular hemorrhage (IVH) that died during the first 3 days of life (mean ± standard deviation).

N	Control Group	Deaths	*p*-Value
	29	5	
Admission Mean (±SD)	5.91 (±3.68)	2.79 (±1.24)	0.311
2nd day Mean (±SD)	4.92 (±2.14)	3.46 (±1.21)	0.266
3rd day Mean (±SD)	3.86 (±1.68)	6.62 (±0.25)	0.023

SD: standard deviation.

## Data Availability

Data are contained within the article.
